# Mechanism of reduced muscle atrophy via ketone body (D)-3-hydroxybutyrate

**DOI:** 10.1186/s13578-022-00826-2

**Published:** 2022-06-20

**Authors:** Jin Chen, Zihua Li, Yudian Zhang, Xu Zhang, Shujie Zhang, Zonghan Liu, Huimei Yuan, Xiangsheng Pang, Yaxuan Liu, Wuchen Tao, Xiaoping Chen, Peng Zhang, Guo-Qiang Chen

**Affiliations:** 1 School of Life Sciences, Tsinghua University, Beijing, 100084 China; 2 National Key Laboratory of Human Factors Engineering, China Astronaut Research and Training Center, Beijing, 100094 China; 3 State Key Laboratory of Space Medicine Fundamentals and Application, China Astronaut Research and Training Center, Beijing, 100094 China; 4 Center for Synthetic and Systems Biology, Tsinghua University, Beijing, 100084 China; 5 MOE Key Lab of Industrial Biocatalysis, Dept of Chemical Engineering, Tsinghua University, Beijing, 100084 China

**Keywords:** Muscle atrophy, Ketone Body, 3-Hydroxybutyrate, Nucleotide synthesis, Protein, Glutamine, Metabolomics

## Abstract

**Background:**

Muscle atrophy is an increasingly global health problem affecting millions, there is a lack of clinical drugs or effective therapy. Excessive loss of muscle mass is the typical characteristic of muscle atrophy, manifesting as muscle weakness accompanied by impaired metabolism of protein and nucleotide. (D)-3-hydroxybutyrate (3HB), one of the main components of the ketone body, has been reported to be effective for the obvious hemodynamic effects in atrophic cardiomyocytes and exerts beneficial metabolic reprogramming effects in healthy muscle. This study aims to exploit how the 3HB exerts therapeutic effects for treating muscle atrophy induced by hindlimb unloaded mice.

**Results:**

Anabolism/catabolism balance of muscle protein was maintained with 3HB via the Akt/FoxO3a and the mTOR/4E-BP1 pathways; protein homeostasis of 3HB regulation includes pathways of ubiquitin–proteasomal, autophagic-lysosomal, responses of unfolded-proteins, heat shock and anti-oxidation. Metabolomic analysis revealed the effect of 3HB decreased purine degradation and reduced the uric acid in atrophied muscles; enhanced utilization from glutamine to glutamate also provides evidence for the promotion of 3HB during the synthesis of proteins and nucleotides.

**Conclusions:**

3HB significantly inhibits the loss of muscle weights, myofiber sizes and myofiber diameters in hindlimb unloaded mouse model; it facilitates positive balance of proteins and nucleotides with enhanced accumulation of glutamate and decreased uric acid in wasting muscles, revealing effectiveness for treating muscle atrophy.

**Graphical Abstract:**

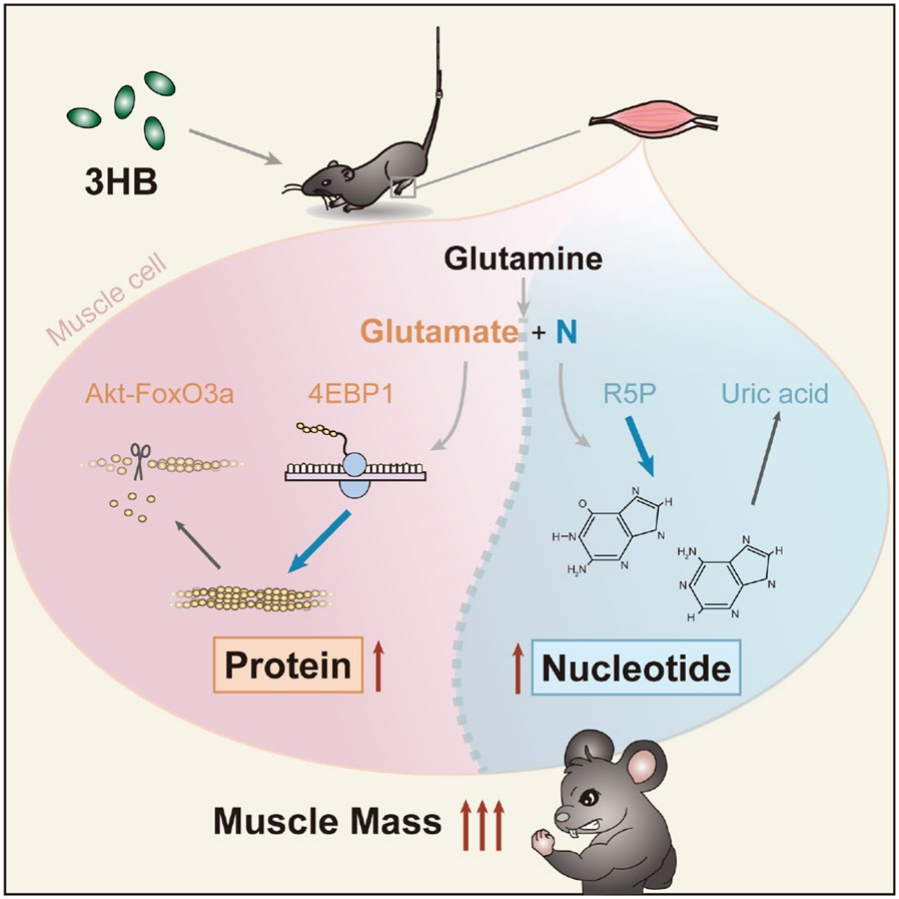

**Supplementary Information:**

The online version contains supplementary material available at 10.1186/s13578-022-00826-2.

## Background

Skeleton muscle accounts for ~ 40% of body weight, playing a vital role in whole-body organ systems, motor performances, and energy metabolism [1]. Inactivity, immobilization, aging, bed rest, and prolonged space flight trigger disuse-induced muscle atrophy, manifesting as muscle weight loss, weakness, physical frailty, and impaired mobility [2]. Excessive loss of muscle mass generally points to the damage of whole-body physiology and increased mortality, negatively impacting prognosis and clinical effectiveness [3]. Although muscle atrophy is a prevailing health problem, there is a lack of clinical drugs or effective therapy. Therefore, the development of safe and effective treatments is of great significance.

Proteins are the most important component of skeletal muscle, serving as gluconeogenesis substrates, playing other nonfuel functional roles such as enzymes, contractile or structural proteins [4]. Considering its role as the largest protein storage place, the preservation of muscle for proteins in skeletal muscle is meaningful for the whole-body capability and metabolism [5]. Accelerated muscle protein degradation primarily occurs as a consequence of the activation of the two major proteolytic pathways, the muscle-specific ubiquitin–proteasomal and the autophagic-lysosomal pathways, both contributing to the loss of muscle mass [6]. Well-accepted ‘atrogenes’, *Atrogin-1* (*Fbxo32*) and *Murf1* (*Trim63*) encoding muscle atrophy F-Box/atrogin-1 (MAFbx) and muscle-specific RING-finger protein 1 (MuRF1) belonging to ubiquitin–proteasomal pathway, are highly expressed in multiple models of muscle atrophy both in mRNA and protein levels [7]. The ubiquitin–proteasomal and the autophagic-lysosomal pathways are both regulated by upstream master transcription factors Forkhead box O-3 (FoxO3/ FoxO3a) directly [8, 9]. Apart from the Akt-Foxo3a signaling pathway regulating muscle protein degradation, the 4E-BP1, a eukaryotic translation initiation factor 4E binding protein 1, is involved in the formation of translation initiation complex which promotes protein synthesis [10].

Nucleotide metabolism is important to support cell proliferation [11, 12]. Nucleotide degradation in atrophic skeletal muscle is a significant phenomenon reducing adenine nucleotides {adenosine triphosphate (ATP), adenosine diphosphate (ADP), adenosine monophosphate (AMP)}, and guanine nucleotides {guanosine triphosphate (GTP), guanosine diphosphate (GDP), guanosine monophosphate (GMP)}, accompanied by the accumulation of degradation products such as inosinic acid (IMP), hypoxanthine, xanthine, and uric acid [13–15]. Glutamine is an important nitrogen donor in muscles supporting the synthesis of proteins, nucleotides and other N-containing components [16, 17].

Prolonged inactivity and insufficient nutrient intake can result in muscle atrophy. Yet hibernators have very little muscle atrophy during hibernation [18, 19]. It becomes interesting to learn if ketone body D-3-hydroxybutyrate (or β-hydroxybutyrate, or 3HB) with high circulating concentrations during hibernationis involved in the regulation of whole-body metabolism and spare muscle proteins [20].

Growing evidence shows an intimate association between muscle protein and 3HB [21]. 3HB and its ester form promote skeletal muscle protein synthesis via decreasing leucine oxidation [22], and activating rapamycin complex 1 (mTORC1) [23]. 3HB was demonstrated as a potent anticatabolic function in human muscle with LPS induced inflammation [24].

3HB is available from the restricted ketogenic diet (KD), the exogenous ingestion of synthesized ketone supplements and the hydrolysis of microbial poly-3-hydroxybutyrate (PHB) [25–28]. It is an energy contributor for cellular activities [25, 29–32]. 3HB utilization is increased in atrophic cardiomyocyte [33, 34], and exogenous 3HB exerts the obvious hemodynamic effects for patients with chronic heart failure (HF) [35, 36]. The conventional ketogenic diet (KD) body supplement and 3HB supplemented to foods or drinks gradually have found applications for treating ﻿neurodegenerative disease such as epilepsy [37, 38], Alzheimer's disease [39, 40], cancer [41, 42], aging [43], atherosclerosis [44], colonic inflammation and carcinogenesis [45], NLRP3-mediated inflammation [46], osteoporosis [47] and enhanced exercise performance [48].

Based on the above studies, we aimed to investigate 3HB as a potential agent for muscle preservation and protection against disuse-induced muscle atrophy.

## Results

### 3HB inhibits soleus muscle weight loss in the hindlimb unloading mouse model

**Fig. 1:**
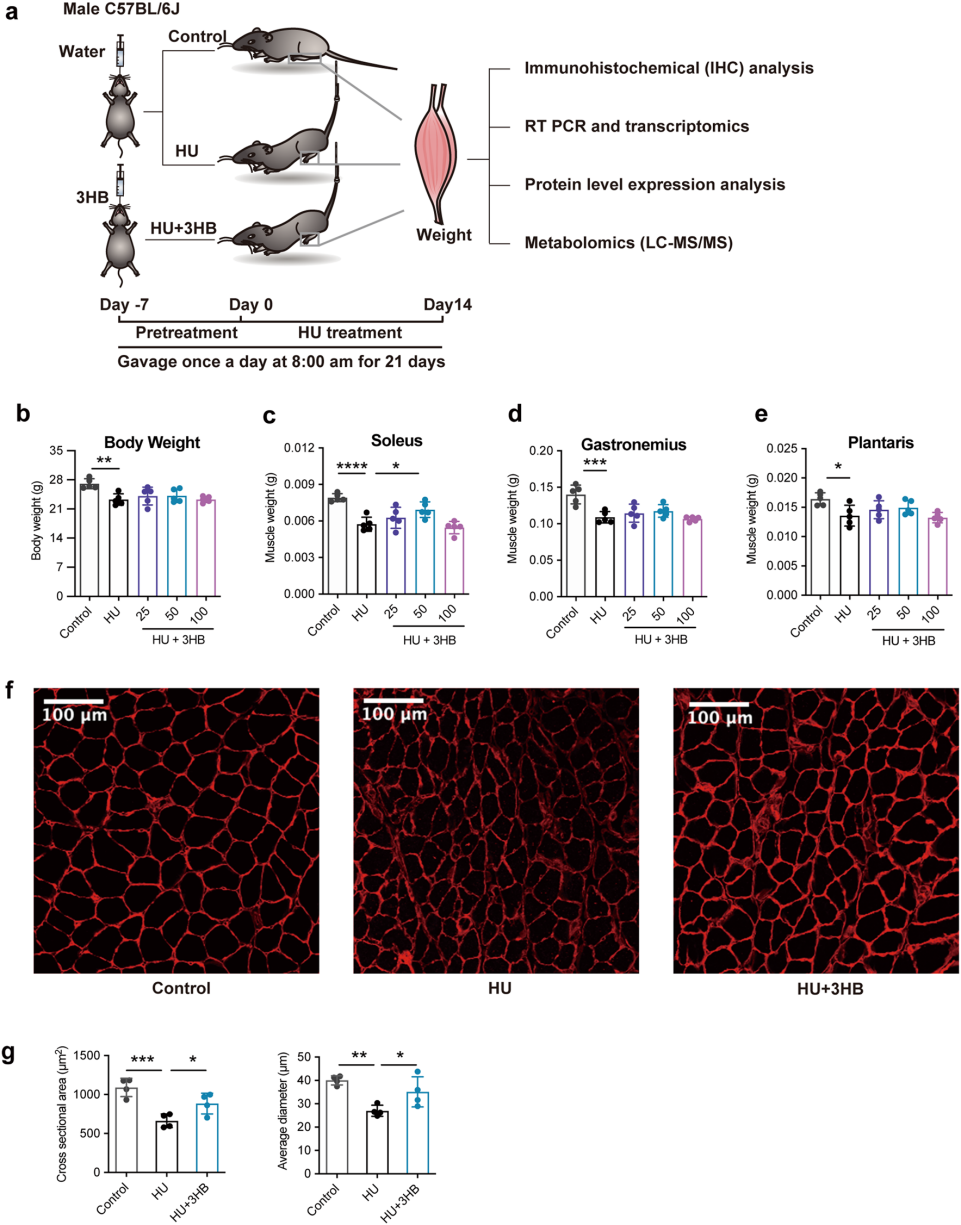
3HB preserves soleus muscle mass in hindlimb unloading mice. **a** Schematic diagram of the development of the hindlimb unloading mouse model. C57BL/6J male mice (8 weeks) were gavage fed with (R)-3-hydroxybutyrate (3HB) 25, 50, and 100 mg/kg body weight once a day for 21 days including 7 days for 3HB pretreatment and 14 days during the hindlimb unloading process. control (ground control group without hindlimbs suspended), HU (hindlimb unloading group), and HU + 3HB group (hindlimb unloading mice fed with 50 mg/kg/days 3HB). Muscle tissues were sampled later for subsequent analyses. **b** Body weight and muscle mass of **c** Soleus, **d** Plantaris, and **e** Gastrocnemius after 14 days of HU treatments (n = 5). **f** Images of immunohistochemical (IHC) analysis in laminin-stained muscles (n = 4 per group). Scale bars = 100 µm. **g** Statistical results of the mean cross-sectional area (CSA) and mean soleus fiber area (n = 4). Error bars are represented as mean ± SD. One-way ANOVA was used for comparison between groups. ****P < 0.0001, ***P < 0.001, **P < 0.01, *P < 0.05, compared with HU mouse group

To investigate whether 3HB can inhibit disuse-induced muscle atrophy, we pretreated mice with different concentrations of 3HB (25, 50, and 100 mg/day/kg body weight) for one week, and then subjected these mice to hindlimb unloading for 2 weeks, during which time, mice were continued to be treated with 3HB via gavage (peroral, PO) (Fig. 1a). Male C57BL/6 J mice showed significant weight loss and atrophy compared with the ground control group. The body weights of the hindlimb unloading (HU) group lost up to 14.11% (**P < 0.01), while the 3HB administration (HU + 3HB) group did not significantly change the body weight compared with the HU group (Fig. 1b).

Masses of soleus, gastrocnemius and plantaris muscles within the HU group decreased by 27.46% (****P < 0.0001) (Fig. 1c), 22.33% (***P < 0.001) (Fig. 1d) and 17.30% (*P < 0.05) (Fig. 1e), respectively, compared to that of the control group. Soleus muscle showed predominantly atrophy in this mouse model. Therefore, changes in soleus were focused on in the subsequent analyses. Among the three 3HB dosages, 50 mg/kg/d 3HB was the optimal dose for preventing the soleus mass loss by up to 20.63% (*P < 0.05) compared within the HU group (Fig. 1c). The amount of food and water uptakes showed no obvious change between the control and HU groups, demonstrating that 3HB did not affect food consumption, further indicating that the muscle atrophy inhibition effects are not attributed to the enhanced food consumption (Fig. 1b).

To investigate whether 3HB affects myofiber size, anti-laminin staining was performed with the soleus muscle cross-sections from the control, HU, and HU + 3HB (50 mg/kg/days) groups, respectively, to visualize the muscle section morphology and to analyze the cross-sectional area (CSA) of individual fibers. Morphological examinations revealed that HU significantly reduced skeletal muscle fiber sizes (Fig. 1f, g), the increase of average myofiber sizes and diameters were observed to be significant after the 3HB treatment (Fig. 1f, g).

### 3HB inhibits upregulation of the ubiquitin–proteasome and autophagy-lysosome in genetic and proteomic levels

**Fig. 2:**
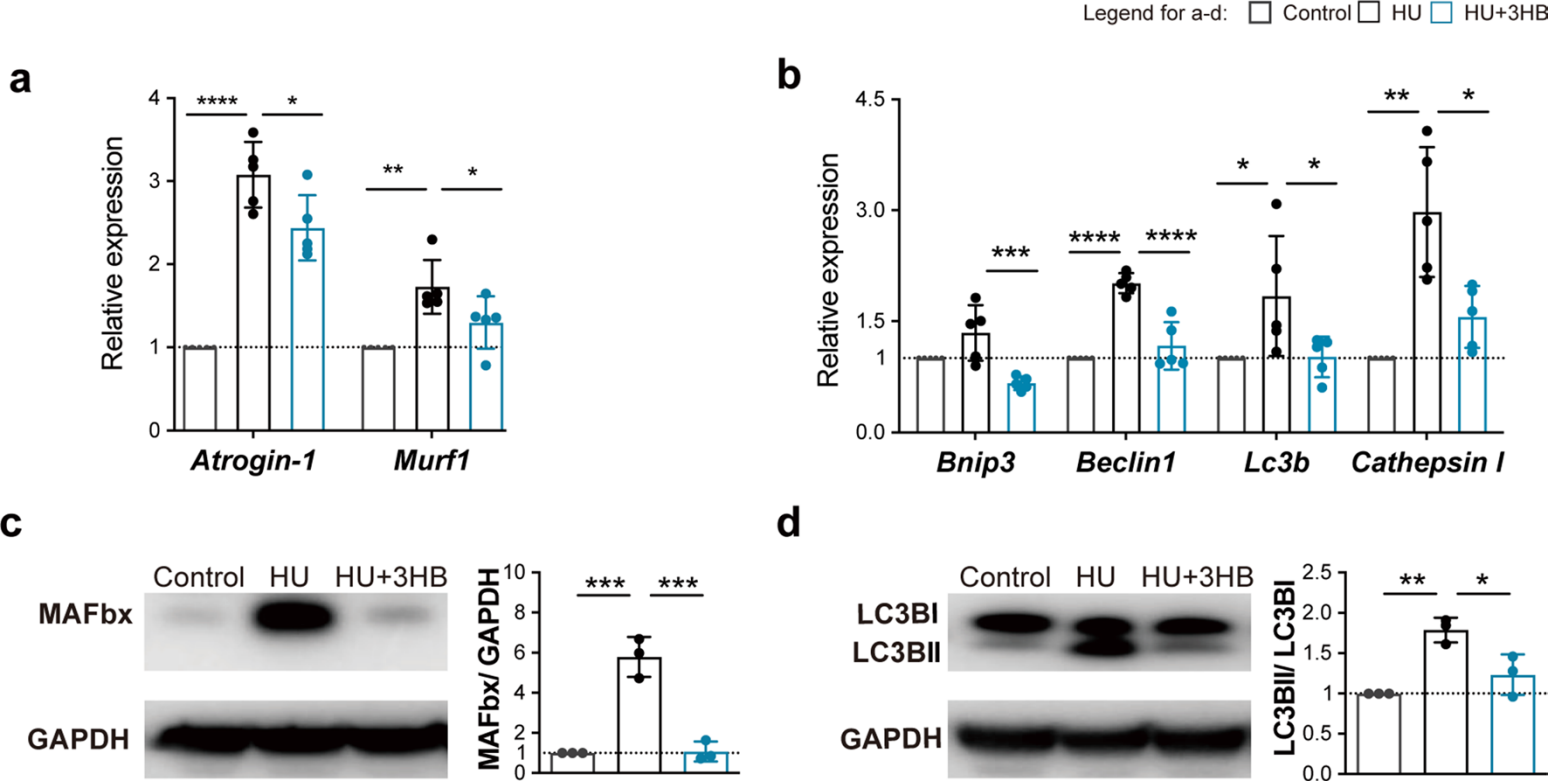
3HB inhibits the upregulation of ubiquitin–proteasome and autophagy-related atrogenes. **a**, **b** Real-time PCR analysis of *Atrogin-1* and* Murf1* **(a)** of *Bnip3, Beclin1, Lc3b and Cathepsin l * (**b**) in soleus muscles of mice in Control (ground control group), HU (hindlimb unloading mouse group), and HU + 3HB (hindlimb unloading mice fed with 50 mg/kg/days 3HB group) (n = 5). The legend box above **b **represents groups of Control (gray), HU (black), and HU + 3HB (blue). Western blot analysis of **c** Ubiquitin protein-Fbx32 (MAFbx) and GAPDH; **d** Autophagy protein-LC3b and GAPDH. The accompanying statistical plots for MAFbx/GAPDH and LC3bII/LC3bI are presented. Immunoblots are representative of the mean values obtained from intensity scans (see also Additional file 1: Fig. S1). Error bars are represented as mean ± SD (n = 3). One-way ANOVA was used for comparison between groups. ****P < 0.0001, ***P < 0.001, **P < 0.01, *P < 0.05, compared with HU mouse group

To further study the mechanism of 3HB on preventing muscle mass loss, quantitative real-time PCR assays and western blots were performed using the soleus muscles of the experimental animals. HU treatments were found to elevate the mRNA expressions of atrogenes (*Atrogin-1* and *Murf1*) and autophagy-related genes *{Lc3b* (*Map1lc3b*), *Beclin1* (*Becn1*), *Bnip3* and *Cathepsin l* (*Ctsl*)} [52]. The up-regulated expression of these genes was found significantly suppressed by 3HB administration (Fig. 2a, b).

To better understand the impact of 3HB on muscle protein degradation systems, MAFbx and LC3bII were investigated for their protein expression levels via western blotting. They were measured as an assessment of the activation of autophagy and the ubiquitin–proteasome system. HU treatment up-regulated MAFbx and LC3bII protein levels (Fig. 2c, d), agreeing with the previous study [6]. 3HB administration significantly decreased the expression of the MAFbx and LC3bII in HU-treated mice by 81.43% (***P < 0.001) and 31.06% (*P < 0.05) (Fig. 2c, d), respectively. These results demonstrated that 3HB inhibits muscle protein breakdown via the regulation of the pathways on ubiquitin–proteasome and autophagy-lysosome systems.

### 3HB prevents muscle protein degradation and maintains proteostasis

To gain a more in-depth insight into the 3HB regulatory roles in the muscle atrophy process, transcriptomic analysis of soleus muscle tissue was conducted via RNA sequencing (RNA-seq). Principal component analysis (PCA) was conducted based on three distributions divided into “Control”, “HU” and “HU + 3HB” groups (Additional file 1: Fig. S3a).

**Fig. 3:**
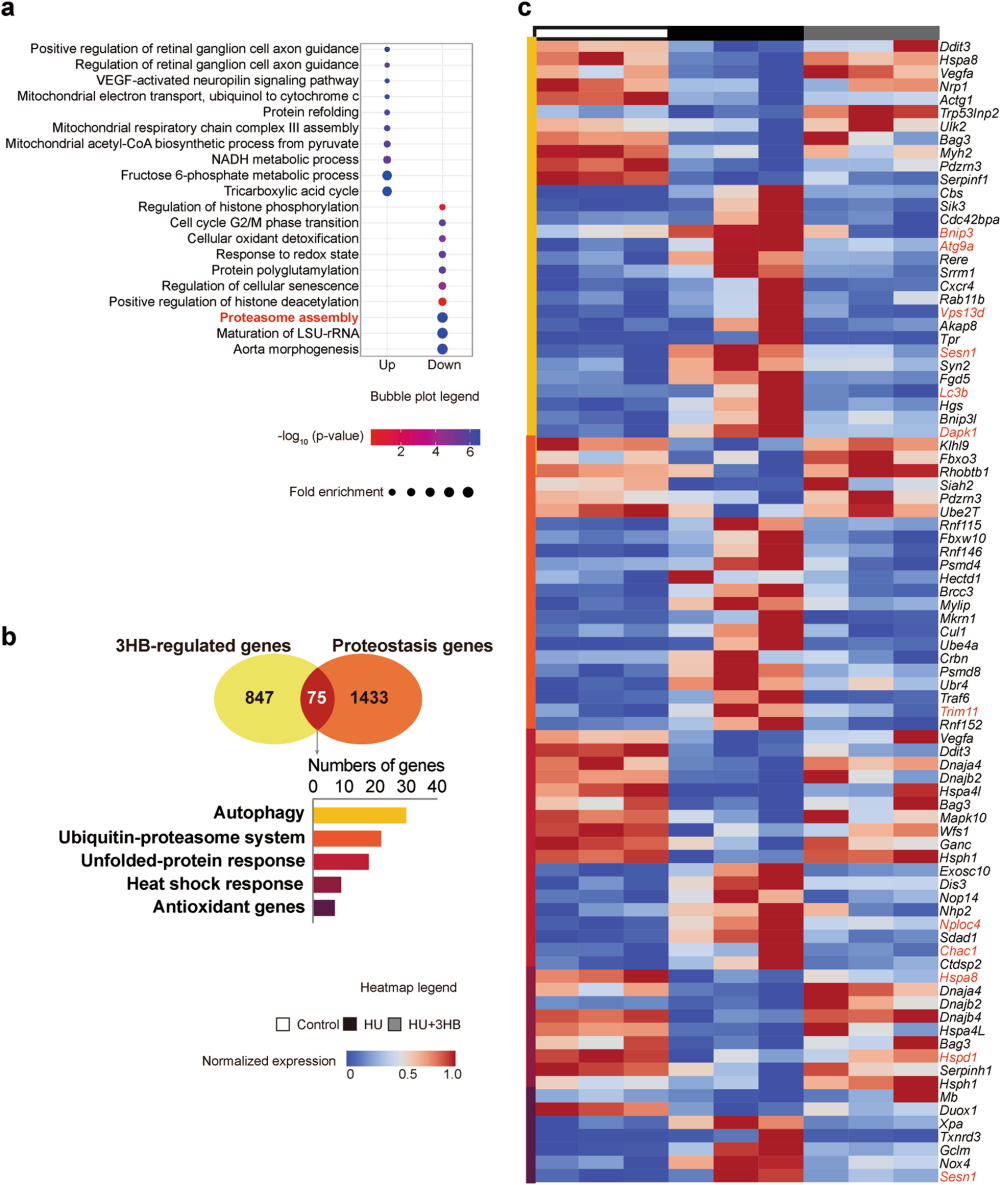
3HB prevents muscle protein degradation and maintains proteostasis. Transcriptomics analysis for soleus muscles of mice in Control (ground control group), HU (hindlimb unloading mouse group), and HU + 3HB (hindlimb unloading mice fed with 50 mg/kg/days 3HB group). n = 3 in each group. **a** Top 10 significantly enriched GO biological processes terms for the up-and down-regulated DEGs in the 3HB-regulated gene set (ranked according to fold enrichment). The bubble size represents gene count containing the amount of DEGs enriched in the pathway, and the bubble color represents the –log_10_ (p-value). **b** Venn diagram showing genes overlapped with the 3HB-regulated genes (defined in Additional file 1: Fig. S2) and a full gene set implicated in proteostasis pathways. **c** The bellowing bar chart shows 3HB-regulated proteostasis genes subdivided into individual pathways. Labels on the right of the heatmap show the gene names. Genes marked in red are the genes selected for separate analysis of FPKM represented gene expression level (Additional file 1: Fig. S3c–g). Detailed information for related gene lists, GO and KEGG analysis are included in Additional file 2: Table S1. The heatmap legend between 0 to 1 is shown in the middle, blue indicates low expression and red indicates high expression

To understand the 731 genes involved in the “3HB-regulated genes” (Additional file 1: Fig. S2), the gene set was used for the analysis of gene ontology (GO) enrichment, followed by studies using the Kyoto Encyclopedia of Genes and Genomes (KEGG) for the upregulated and downregulated genes based on the fold enrichment values (Fig. 3a). The top 10 GO terms were ranked based on the fold enrichments.

Pathways including “mitochondrial electron transport (ubiquinol to cytochrome c)”, “mitochondrial respiratory chain complex III assembly”, “mitochondrial acetyl-CoA biosynthetic process from pyruvate”, “tricarboxylic acid cycle” and “NADH metabolic process” were enriched during the 3HB related up-regulated biological process (BP) (Fig. 3a). While in the 3HB up-regulated KEGG pathways, “oxidative phosphorylation”, “citric acid cycle (TCA cycle)”, “pyruvate metabolism”, “galactose metabolism”, “central carbon metabolism in cancer”, “alanine, aspartate and glutamate metabolism” were all enriched (Additional file 1: Fig. S3b). These energy metabolism-related terms including central carbon metabolism, oxidative phosphorylation, and mitochondrial electron transport chain (ETC) point to a protective metabolic regulation of 3HB in skeleton muscles.

Among the significantly enriched down-regulated GO biological processes (BP), 3HB showed the protective efficacy against “proteasome assembly”, “cellular oxidative detoxification”, “responses to a redox state” and “regulation of cellular senescence” (Fig. 3a). Hindlimb unloading (HU) studies on model mice induced atrophy accompanied with the occurrence of the metabolic switch controlled by tightly coordinated transcriptional and epigenetic changes, allowing to easily understand “regulation of histone phosphorylation”, “protein polyglutamylation”, “positive regulation of histone deacetylation” and “maturation of LSU-rRNA” that were also included in 3HB regulation to the normal physiological state (Fig. 3a). These results indicated that protein degradation and cell stress-responsive genes were upregulated activated by HU, and 3HB administration significantly inhibited the atrophic responses.

To unequivocally define the coordination of metabolic state with proteostasis system and the specific role for 3HB anti-amyotrophy in muscle protein, overlapping “3HB-regulated genes involving the complete set of genes encoded in proteostasis pathways were analyzed (Fig. 3b). 30, 22, 18, 9, and 7 overlapped genes in the whole list containing genes of “autophagy”, “ubiquitin–proteasome system”, “unfolded-protein response”, “heat shock response” and “antioxidant genes” were considered, respectively (Fig. 3b). These overlapped genes behaved with reverse tendency after the 3HB treatments, as evidenced by the relative expression abundance of 75 genes shown in the heat map (Fig. 3c). “3HB-regulated genes” were highly enriched in two classic muscle atrophy pathways related to “autophagy” and “ubiquitin–proteasome system”, they play a major role in protein homeostasis during the muscle atrophy process as supported by previous studies [7, 52].

For example, *Map1lc3b* (also called *Lc3b*), and *Bnip3* were enriched in the “autophagy” overlapped part, confirming the quantitative real-time PCR results (Fig. 2b). In addition, genes encoding transmembrane protein ATG9 (*Atg9a*) [53], a ubiquitin-binding protein able to regulate mitochondrial fission (*Vps13d*) [54], and death-associated protein kinase 1 (*Dapk1*) [55], were also found enriched in “autophagy” overlapped part, which is essential for the formation and the transport of autophagosomes in the signal pathways of cell survival and apoptosis. In the “ubiquitin–proteasome” system, *Trim11* was enriched encoding E3 ubiquitin-protein ligase TRIM11, which promotes the degradation of insoluble ubiquitinated proteins [56]. In the “unfolded-protein response” pathway, genes encoding ubiquitin recognition factor responsible for exporting the misfolded proteins from the ER to the cytoplasm (*Nploc4*) [57], a pro-apoptotic component downstream the ATF4-ATF3-CHOP cascade (C*hac1*) [58], were included. While the “antioxidant genes” encoding antioxidant sestrin (*Sesn1*) functioning to prevent muscle atrophy [59], were also enriched for “autophagy”. The “heat shock response” genes such as *Hspa8* and *Hspd1* encoding heat shock proteins (HSPs) that help refold proteins and restore muscle function were found enriched [60, 61]. The regulation of 3HB for the expression level of each selected gene were assessed by fragments per kilobase of exon per million fragments mapped (FPKM), respectively (Additional file 1: Fig. S3c–g). Genes *Atg9a*, *Vps13d,* and *Chac1* were observed to have similar results between quantitative real-time PCR assays and the transcriptomic data (Additional file 1: Fig. S3h).

The above results suggest that 3HB regulates proteostasis and maintains protein content in skeletal muscles via a comprehensive portfolio of regulators belonging to “autophagy”, “ubiquitin–proteasome”, “unfolded-protein response”, “heat shock response” and “antioxidative genes”.

### 3HB influences protein metabolism by regulating Akt/FoxO3a and mTOR/4E-BP1 pathways

**Fig. 4:**
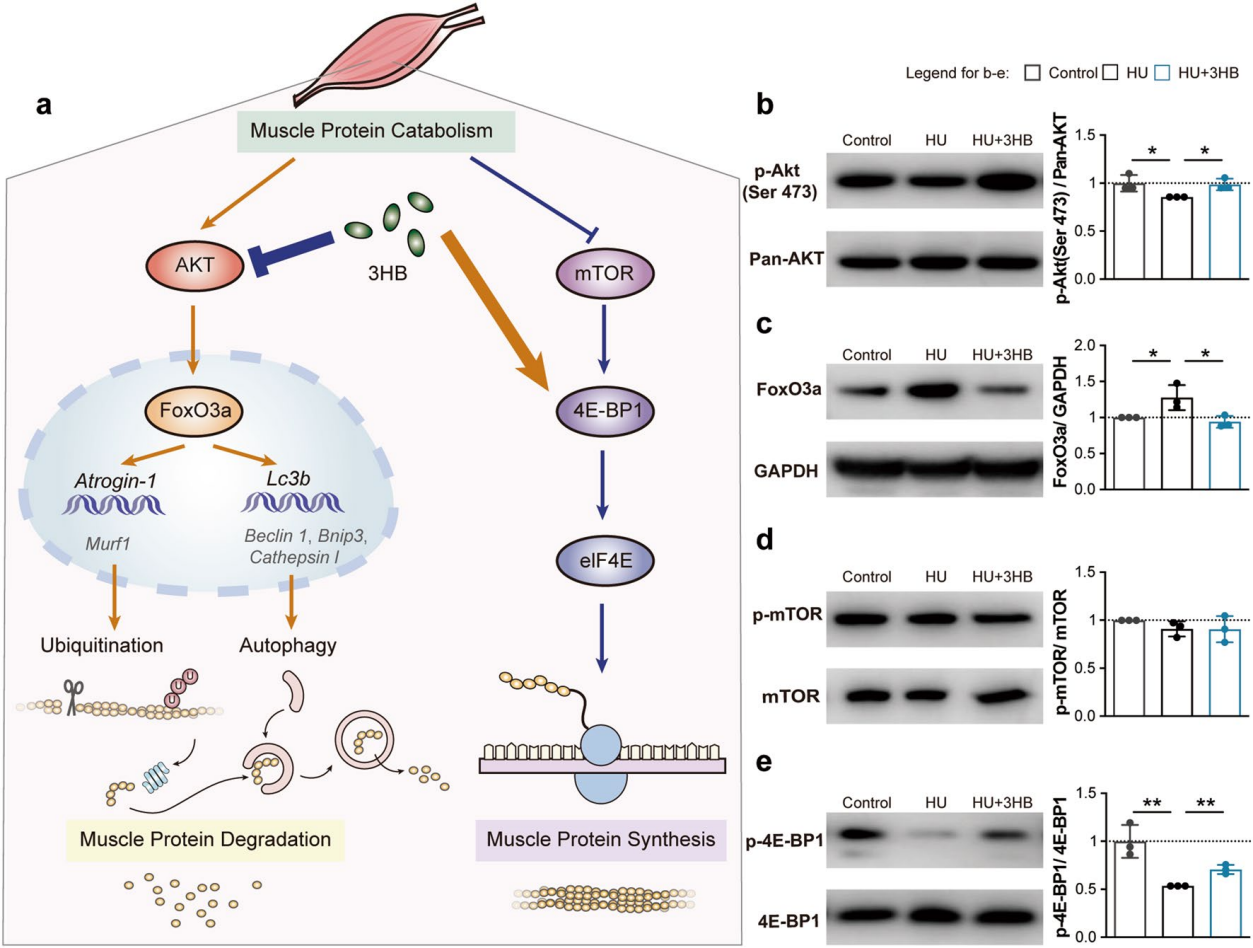
3HB influences protein metabolism by regulating Akt/FoxO3a and mTOR/4E-BP1 pathways. Protein expression via the protein homeostasis signaling pathway revealed by western blot of the soleus muscles of mice in groups of Control (ground control group), HU (Hindlimb unloading mouse group), and HU + 3HB (hindlimb unloading mice fed with 50 mg/kg/days 3HB group). The legend box on the top right corner represents different groups. Control (gray), HU (black) and HU + 3HB (blue). **a** Schematic presentation of 3HB regulation in muscle protein catabolism. Western blot analysis of **b** phosphorylation levels of Akt (S473) and pan-AKT; **c** FoxO3a and GAPDH; **d** phosphorylation levels of mTOR (S2448) and mTOR; and **e** phosphorylation levels of 4E-BP1 (T37/46) and 4E-BP1. The accompanying statistical plots for p-Akt (S473)/pan-AKT; FoxO3a/GAPDH; p-mTOR(S2448)/mTOR; and p-4E-BP1(T37/46)/4E-BP1 are presented on the side (see also Additional file 1: Fig. S4). Immunoblots are representative of the mean value obtained from intensity scans. Error bars are represented as mean ± SD (n = 3). For **b**, **e**, an unpaired, two-tailed Student’s t-test was used. For **c**, one-way ANOVA was used for comparison between groups. ****P < 0.0001, ***P < 0.001, **P < 0.01, *P < 0.05, compared with HU mouse group

To further understand the inhibitory effect of 3HB on protein degradation via ubiquitin–proteasome and autophagy-lysosome systems, the protein expression level of phosphorylation of the upstream Akt/FoxO3a pathway was examined, together with the mTOR/4E-BP1 pathway of protein synthesis for comprehensive consideration of maintaining cellular protein homeostasis (proteostasis) (Fig. 4a).

Akt phosphorylates transcriptional factors FoxO3a, leading to nuclear export and cytoplasm retention of phosphorylated FoxOs, thus affecting their transcriptional functions [62]. Phosphorylation levels of the Akt at S473 were reduced by HU treatment, which was increased by 11.54% (*P < 0.05) in 3HB treated mice compared with the hindlimb unloaded ones (Fig. 4b). The down-regulated expression of Akt phosphorylation was concomitant with a 26.44% (*P < 0.05) decrease in Foxo3a expression (Fig. 4c).

For the muscle protein anabolism, a not significant change was observed in mTOR phosphorylation at S2448 among the three groups (Fig. 4d). The phosphorylation level of 4E-BP1 at thr371/46 in atrophied soleus was decreased by 46.34% (**P < 0.01) compared with that of the control soleus, and 3HB administration up-regulated the phosphorylation level by 31.85% (**P < 0.01) (Fig. 4e). The significant increase of 4E-BP1 phosphorylation at thr37/46 indicates the activation of translation initiation and promotes the protein synthesis process.

The above results suggest that 3HB can partially compensate for decreased protein synthesis and increased protein degradation resulted from HU treatment via Akt/FoxO3a and mTOR/4E-BP1 pathways.

### 3HB participates in nucleotide metabolism and reduces uric acid accumulation in atrophied muscle

To explore the functional consequences of 3HB treatment on the metabolic properties of skeletal muscle, the targeted and untargeted metabolomic analysis of soleus muscles was collected 14 days after 3HB or water treatments to the HU mouse model.

**Fig. 5:**
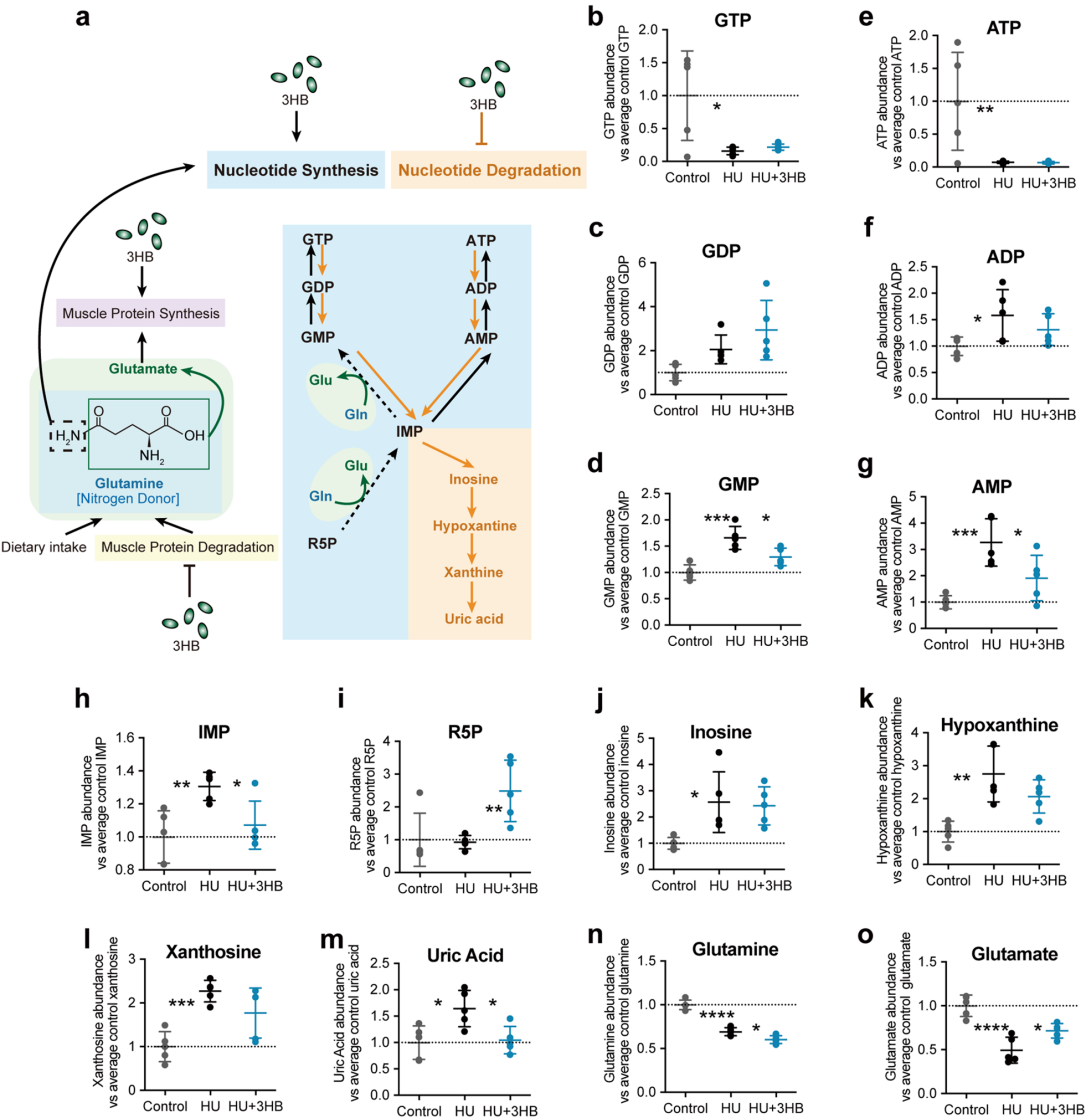
3HB participates in nucleotide metabolism and reduces uric acid accumulation in atrophied muscle. Effects of 3HB on skeletal muscle metabolism in soleus muscles of mice in Control (ground control group), HU (hindlimb unloading mouse group), and HU + 3HB (hindlimb unloading mice fed with 50 mg/kg/days 3HB group). For each identified metabolite, the raw data of peak area for each sample in the Control, HU, and HU + 3HB groups were normalized to the average peak area of the control group. **a** Schematic presentation of 3HB regulation in nucleotide metabolism in skeletal muscle [14]. The relative metabolite abundance for each metabolite between groups. **b** Relative abundance of guanosine triphosphate (GTP). **c** Relative abundance of guanosine diphosphate (GDP). **d** Relative abundance of guanosine monophosphate (GMP). **e** Relative abundance of d-ribulose-5-phosphate (R5P). **f** Relative abundance of adenosine triphosphate (ATP). **g** Relative abundance of adenosine diphosphate (ADP). **h** Relative abundance of adenosine monophosphate (AMP). **i** Relative abundance of inosinic acid (IMP). **j** Relative abundance of inosine. **k** Relative abundance of hypoxanthine. **l** Relative abundance of xanthine. **m** Relative abundance of uric acid. **n** Relative abundance of glutamate. **o** Relative abundance of glutamine. Error bars are represented as mean ± SD (n = 5). one-way ANOVA was used for comparison between groups. ****P < 0.0001, ***P < 0.001, **P < 0.01, *P < 0.05, compared with HU mouse group. Raw data of identified metabolites and normalized data for nucleotide metabolism are listed in Additional file 3: Table S2

To verify the inhibition function of 3HB in “RNA degradation” which was shown in the 3HB down-regulated KEGG pathway (Additional file 1: Fig. S3b), the metabolomics analysis in nucleotide metabolism was conducted. Our results revealed that GTP and ATP and content of atrophied soleus in the HU group were significantly reduced by 92.69% (**P < 0.01) and 83.92% (*P < 0.05), respectively, relative to that in the control group (Fig. 5b, e). Increased nucleotide degradation was accompanied by the accumulation of GDP (Fig. 5c), ADP (Fig. 5f), GMP (Fig. 5d), AMP (Fig. 5g), and downstream products IMP (Fig. 5h), inosine (Fig. 5j), hypoxanthine (Fig. 5k), xanthine (Fig. 5l) and uric acid (Fig. 5m).

Uric acid, the end product of purine metabolism, was accumulated by 64.60% (*P < 0.05) more in the atrophied soleus compared with the normal control soleus (Fig. 5m). 3HB treatments showed down-regulation in the purine nucleotide degradation pathway with a significant tendency for reducing intermediates (Fig. 5c–h, j-i) and end-products, a remarkable reduction in uric acid level by 36.46% (*P < 0.05) was observed after 3HB treatments (Fig. 5m). The content of nucleotide synthesis precursor, ribose-5-phosphate (R5P), was increased by 167.99% (**P < 0.01) after 3HB treatment (Fig. 5i). The process from R5P to IMP and IMP to GMP requires the transformation of glutamine to glutamate (Fig. 5a) [16]. Glycine, which provides all its carbon and nitrogen atoms to purine rings important for cell proliferation [63], was observed up-regulated by 3HB (Additional file 1: Fig. S5a). The increased nucleotide synthesis and decreased nucleotide degradation indicate that 3HB exerts the function to promote cell proliferation at the nucleotide metabolism level.

It was found that glutamine and glutamate levels in the atrophied soleus were decreased by 30.79% (****P < 0.0001) (Fig. 5n) and 50.44% (****P < 0.0001) (Fig. 5o), respectively, compared to that in the control group. 3HB treatment showed up-regulating glutamate level by 44.59% (*P < 0.05) (Fig. 5o), while down-regulating glutamine level by 13.11% (*P < 0.05) (Fig. 5n).

Major inhibitory neurotransmitter gamma-aminobutyric acid (GABA) (Additional file 1: Fig. S5c), N-acetyl-aspartyl glutamate (NAAG) (Additional file 1: Fig. S5b), and acetylcholine (Additional file 1: Fig. S5d) were observed decreased via 3HB administration. At the same time, 3HB was revealed to inhibit the accumulation of L-tryptophan (Additional file 1: Fig. S5k) and its metabolite indole-acrylic acid (IA) (Additional file 1: Fig. S5l) in atrophied soleus.

3HB administration failed to restore the glycolytic metabolism (the content of lactic acid) compared with the control group (Additional file 1: Fig. S5j). Similarly, changes in metabolite contents in the TCA cycle were not significant among the three groups (Additional file 1: Fig. S5e–j).

It can be concluded that skeletal muscle undergoes metabolic adaptability in the disuse-induced muscle atrophy model, 3HB presence improves the synthesis of proteins and nucleotides, enhancing glutamate accumulation and decreasing uric acid accumulation, contributing to maintaining the metabolic balance in skeletal muscles.

## Discussion

When disuse-induced muscle atrophy and other metabolic disorders occur, muscle proteins are always degraded and adapted muscle mass to different pathophysiological conditions. This study identified 3HB to be able to preserve muscle mass and fiber areas as it helps the maintenance of muscle proteins (Fig. 1c, f, g). 3HB plays a crucial role in the regulation of the Akt-FoxO3a pathway (Fig. 4b, c), inhibiting downstream ubiquitin–proteasome (Fig. 2a, c) and the autophagy-lysosome systems (Fig. 2b, d). The multilevel integration in unfolded-protein responses, heat shock responses, and antioxidant responses contribute to the 3HB catabolic inhibition (Fig. 3b, c). Increased protein synthesis was confirmed by decreased 4EBP1 phosphorylation (Fig. 4e) and increased content of glutamate (Fig. 5o). Glutamate is used for protein synthesis in almost all living beings and glutamine is the substrate used for nucleotide synthesis, the two-way conversion between glutamine and glutamate is involved in the maintenance of cell integrity and cellular functions [17].

Muscle glutamine was released responding to the disuse and the decreased glutamine storage represents the significant muscle catabolic state (Fig. 5n ). The source of glutamine includes the dietary intake and the muscle protein catabolism [17, 64], the latter was verified to be inhibited by 3HB (Figs. 2a, 3, 4b-c). The demands of the conversion of glutamine to glutamate are elevated in the nucleotide synthesis process [16], which was evidenced by the increased R5P and decreased uric acid (Fig. 5i, m) in nucleotide metabolism. This reaction results in elevated demands of the conversion of glutamine to glutamate. Thus, combining the decreased source and increased utilization, it is not difficult to understand why 3HB administration decreases the content of glutamine (Fig. 5n) and simultaneously increased the accumulation of glutamate (Fig. 5o). The surprising decrease of uric acid in atrophied muscle via 3HB leads us to consider the association between muscle atrophy and gout, and this may provide a clue for the drug widely used to treat gout that can attenuate muscle atrophy [65]. 3HB was reported to block NLRP3 inflammasome [46] and this deactivation in neutrophil caused by ketone diet relieves gout flares [66]. This study demonstrates that the direct 3HB treatment can be an effective way for treating patients with muscle atrophy and/or gout due to its beneficial metabolic regulation in musculoskeletal disorders.

Considering that 3HB can cross the blood–brain barrier, the possible association between a motor neuron and skeletal muscle cannot be excluded [67]. While muscle-derived glutamate has been reported to direct reallocation of energy substrates and acts as an important metabolite in the gut-muscle-fat axis [68], the well-known function of glutamate is an excitatory neurotransmitter responsible for the control of locomotion [69]. Glutamate plays a signaling role in the vertebrate neuromuscular junctions [70]. Upon stimulation, glutamate can be produced by N-acetyl-aspartyl-glutamate (NAAG), the most abundant and wide-distributed neuropeptide in the central nervous system of mammals [71, 72]. NAAG can modulate the release of acetylcholine (Ach) from motor nerve endings [72]. Glutamate also serves as a precursor for the synthesis of gamma-aminobutyric acid (GABA), an inhibitory neurotransmitter that acts as a muscle relaxant [73]. The intramuscular content of the most four prevalent neurotransmitters was significantly altered by 3HB. NAAG, GABA, and Ach (Additional file 1: Fig. S5b-d), were significantly decreased while glutamate was observed increasing intriguingly (Fig. 5o). The increased consumption of the precursor L-tryptophan (Additional file 1: Fig. S5k), may provide more serotonin (5-hydroxytryptamine, 5-HT), possible evidence for improving the depression symptoms [74] and may relieve the incapacitating myalgia by reducing the cytotoxicity probably caused by L-tryptophan in eosinophilia-myalgia syndrome epidemic [75].

3HB is assumed to exert its functions via the post-translational modifications (PTMs), namely, “β-hydroxybutyrylation” on histone and non-histone proteins [76–80], “sense” metabolic changes caused by the disused and modulated downstream gene expression. Although 3HB is an important fuel molecule with high energetic efficiency, the current approach is not precise enough to observe the reallocation of energy substrates in terms of glycolysis (Additional file 1: Fig. S5j), fatty acid oxidation (Additional file 3: Table S2), and TCA cycle (Additional file 1: Fig. S5e-i). These results further revealed that 3HB regulates muscle protein via different mechanisms during exercises under healthy and muscle atrophy conditions [48].

A recent study suggests that the hibernators re-obtain nitrogen source from urea and reincorporate nitrogen for the synthesis of amino acids and proteins [81]. We hypothesize that 3HB plays a role to increase nitrogen utilization from harmful metabolic wastes including urea and uric acid in hibernating animals. More studies have confirmed the inhibitory function of reduced muscle mass by ketogenic diet and R/S-1,3‐butanediol acetoacetate diester in aging, and cancer anorexia cachexia syndrome (CACS) [82, 83]. Despite the long history of known positive effects of the ketogenic diet, the high-fat, low carbohydrate diet needs rigorously medically supervision due to poor patient compliance and struggle to adherence with potential risks of hyperlipidemia and fatty liver disease [25]. The ketone bodies ester derivative used in CACS is hydrolyzed in the liver to release bi-chiral 3HB, and the L-isomer 3HB is the unnatural form of ketone body in mammals with potential harms to the human body. Besides the limited therapy, the multifactorial CACS model includes cancer, cachexia, systemic inflammation, anorexia, and anemia, providing only indirect evidence of anti-amyotrophy function in confounding and comorbid environments. The present study provides for the first-time direct evidence that 3HB inhibits muscle atrophy in the one-factor model, extending the application of ketogenic diet (KD) and exogenous ketone (ketogenic) supplements for healthy purposes.

3HB, a natural molecule, can find applications in the muscle atrophy area without side effects. From an endogenous small-molecule point of view, these results indicate that 3HB can attenuate disuse-induced muscle atrophy and provide an option for possible nutritional supplements to increase prognosis and life expectancy. 3HB may also pose tractable implementation for the weight loss ones to save muscle mass in the fat-only loss condition and those bodybuilders desiring to get stronger muscular fitness and more muscle mass [48, 84]. Our mouse model was found simulating the skeletal muscle atrophy in a weightless environment, which share similar pathophysiological changes and metabolic mechanisms with senescent state [85, 86]. Therefore, our study lays a beneficial foundation for future space explorers and the aging populations. 3HB can serve as the hydrolysis products of biocompatible PHA biomaterials [87–89]. 3HB related biomaterials can be used as implant biocompatible scaffolds supporting tissue regeneration in tissue engineering [90–92], the application in nanoparticle platform for drug delivery has also been revealed [93, 94]. Our study provides values for the potential benefits for subsequent production of protein and nucleotides. Studies on engineered microbes and synthetic biology for the production of 3HB may have practical applications and high market values in the healthcare field [95, 96]. 3HB deserves more studies to improve physical functions and quality of life in the best interest of mankind.

## Conclusions

For the first time, the alleviation effect of 3HB was evaluated in the atrophied model animals caused by hindlimb unloading. Direct evidence was provided for the effective inhibition of disused muscle atrophy in the one-factor model. 3HB, a natural molecule, extends the application of ketogenic diet (KD) and exogenous ketone (ketogenic) supplements for healthy muscle purposes and excludes its potential health risk. The functional activation of the transformation and utilization for glutamine as well as the multi-dimensional regulation of the proteins and nucleotides turnover provides an option for possible nutritional supplements to patients with muscle atrophy.

## Methods

### Mice and their 3HB treatments

Animal studies were performed using 8-weeks-old C57BL/6J male mice purchased from Vital River Laboratory Animal Technology Co (Beijing, China). All mice were housed in Tsinghua animal house, an adequately ventilated temperature-controlled rodent-housing system with free access to food and water.

Mice were weighed and grouped into three clusters of 5 mice each: Control (ground control group), HU (hindlimb unloading mouse group), and HU + 3HB (hindlimb unloading mice fed with 3HB group). Before the mice were suspended with their hindlimbs, R-3-hydroxybutyric acid sodium salt (3HB, Sigma-Aldrich, USA) or an equal volume of deionized water were administrated in the pretreatment period for 7 days, then continued for another 14 days during the hindlimb unloading model construction. 3HB and deionized water were administrated for 21 days by oral gavage once a day at 8:00 a.m.

### Hindlimb unloading induced muscle atrophy model

According to the protocol by the National Aeronautics and Space Administration (NASA) Ames Research Center [49], we used a classic hindlimb unloading mouse model to simulate the weightless environment, the mice were suspended via tail tied to the top of the single cage as previously described [50]. In this model, hindlimbs were not allowed to contact the ground and forelimbs were free to move on the ground allowing access to water and food ad libitum. This model simulating weightless situations and inactivity of mice caused muscle atrophy in their hindlimbs.

Soleus, plantaris, and gastrocnemius were carefully collected, weighted, and frozen immediately in an isopentane/liquid nitrogen and stored in liquid nitrogen until subsequent analyses.

### Immunohistochemical analysis

Immunohistochemical (IHC) analysis was performed using standard techniques as described [51]. Mouse soleus muscle sections (10 μm) were prepared and subjected to immunofluorescence staining with anti-laminin (Abcam, ab11575, USA) and Goat anti-Rabbit IgG (H + L) Highly Cross-Adsorbed Secondary Antibody, Alexa Fluor 594 (Invitrogen, A11037, USA). Images were acquired using a TSC SP5 Leica confocal microscope, with an HCX PL APO CS 40 × /1.3 NA oil CS objective lens (Germany), equipped with LAS AF Lite software. Myofiber size and diameter were obtained by measuring the cross-sectional area (CSA) and the distance between the myofiber manually quantified using *ImageJ* software.

### RNA isolation, reverse transcription, and real-time PCR analysis

Total RNA extraction was prepared from mouse soleus muscles using TRIzol reagent (Thermo Fisher, 15596026, USA) and the cDNAs were generated by reverse transcription kit (Qiagen, 205311, Germany). qPCR was performed using the standard protocol in ABI 7500 Fast Real-Time PCR System (Applied Biosystems™ 7500, Thermo Fisher, USA) using SYBR Green Master Mix (Applied Biosystems, A25743, USA). 18 s Mouse rRNA normalizes the expression of mRNA for genes of interest. Primers used for qPCR are listed (Table 1).

**Table 1:** Summary of the primers used in this study

Name	Primer
*Atrogin-1-F*	5′-CTTCAAAGGCCTCACGATCAC-3′
*Atrogin-1 -R*	5′-CAGCCTCTGCATGATGTTCAG-3′
*Murf1-F*	5′-CAACCTGTGCCGCAAGTGT-3′
*Murf1-N-R*	5′-GGGATTGCCACAGAGGATTAGA-3′
*18 s Mouse rRNA-F*	5′-CCAGAGCGAAAGCATTTGCCAAGA-3′
*18 s Mouse rRNA-R*	5′-TCGGCATCGTTTATGGTCGGAACT-3′
*Cathepsin l-F*	5′-ACAGAAGACTGTATGGCACGA-3′
*Cathepsin l-R*	5′-GTATTCCCCGTTGTGTAGCTG-3′
*Lc3b-F*	5′-TTATAGAGCGATACAAGGGGGAG-3′
*Lc3b-R*	5′-CGCCGTCTGATTATCTTGATGAG-3′
*Becn1-F*	5′-ATGGAGGGGTCTAAGGCGTC-3′
*Becn1-R*	5′-TGGGCTGTGGTAAGTAATGGA-3′
*Bnip3-F*	5′-CTGGGTAGAACTGCACTTCAG-3′
*Bnip3-R*	5′-GGAGCTACTTCGTCCAGATTCAT-3′
*Nploc4-F*	5′-TGAAGAGGATCACAGCTACGA-3′
*Nploc4-R*	5′-CGCCATGCTTGATTTTTAGCAA-3′
*Chac1-F*	5′-CTGTGGATTTTCGGGTACGG-3′
*Chac1-R*	5′-CCCCTATGGAAGGTGTCTCC-3′
*Atg9a-F*	5′-CCGAGGGGAGCAAATCACC-3′
*Atg9a -R*	5′-TAGTCCACACAGCTAACCAGG-3′

### Transcriptome sequencing and analysis

The transcriptome sequencing processes were performed by the OE Biotech Co., Ltd. (Shanghai, China). A cDNA library was constructed using qualified soleus muscle samples. RNA sequencing was performed using Illumina HiSeqTM 2500 sequencer to sequence 125 bp or 150 bp. The differential expressed genes (DEGs) were screened with the threshold P-value < 0.05 in bioinformatics analyses. PCA diagram was drawn with the filtered set of genes normalized by fragments per kilobase of exon per million fragments mapped (FPKM) and using the python-based sklearn plugin. Volcano plots were drawn using the R package DESeq2. Gene ontology (GO) and Kyoto Encyclopedia of Genes and Genomes (KEGG) pathway analysis were performed using DAVID (https://david.ncifcrf.gov) to annotate the functions of DEGs. The top 10 enriched GO terms of up-regulated and down-regulated DEGs are presented ranked according to fold enrichment, the significantly changed KEGG pathways were filtered by P-value < 0.05. Original data are listed in Additional file 2: Table S1. Source transcriptomic data are available through Sequence Read Archive (SRA) submission: SUB10968179. Other original data are available upon request.

### Metabolomic analysis

50 mg muscle tissue samples were extracted by 80% (v/v) HPLC‐grade after homogenization, then Speedvac (Thermo Savant, NY, USA) was used to dry the supernatant of the homogenates. Targeted and untargeted metabolomics analysis was performed on the Metabolomics and Lipidomics Center at Tsinghua-National Protein Science Facility (Beijing).

Targeted metabolomics was conducted using a TSQ Quantiva Triple Quadrupole mass spectrometer coupled with Ultimate 3000 UHPLC system (Thermo, CA). Metabolites related to central carbon metabolism including glycolysis pathway, TCA cycle, amino acids, and purine metabolism were analyzed using ion-pairing chromatography with Hydro-RP C18 column (2.0 × 100 mm, Phenomenex). Solvent A is 10 mM tributylamine and 15 mM acetate in water; Solvent B is 100% methanol. All data were acquired in positive–negative ion switching mode. Q1 and Q3 were set with 0.7-FWHM-resolution windows. The source voltage was 3.5 kV for positive and 2.5 kV for negative ion mode. The instrument parameters were optimized as follows: spray voltage, capillary temperature, heater temperature, sheath gas flow rate and auxiliary gas flow rate were 3000 V, 320 °C, 300 °C, 35 arb, and 10 arb, respectively. Metabolite identification was achieved using Trace Finder software (Thermo, CA) with a home-built database containing ~ 300 compounds.

Untargeted metabolomics was performed using QExactive Orbitrap mass spectrometer with Ultimate 3000 UHPLC system (Thermo, CA). The instrument parameters were optimized as follows: The spray voltage mode was 3.5 kV in positive mode. MS with mass range of m/z 70–1050 was applied. Data were acquired using 70,000 MS resolution and 17,500 MS/MS resolution. The sheath gas flow rate and aux gas flow rate were 35 arb and 8 arb, respectively. BEH Amide column (2.1 × 100 mm, Waters) was applied for LC separation. Metabolite identification was performed using TraceFinder 3.2 (Thermo, CA) with a home-built database containing standard MS/MS spectra of over 1500 metabolites. Identified metabolites are listed in Additional file 3: Table S2. For relative quantitation, the raw data of peak areas for each sample in the Control, HU, and HU + 3HB groups were normalized to the average peak area of the control group.

### Western blot analysis

40 µg total protein lysates were prepared by enhanced RIPA lysis buffer (Biorigin, China) with phosphatase inhibitor cocktail (Biorigin, China), protease inhibitor (Biorigin, China), PMSF (Biorigin, China) and then resolved on NuPAGE 4–12% Protein Gel (Invitrogen, NP0321bOX, USA) and transferred at 200 mA for 90 min onto polyvinylidene difluoride membrane (0.22 μm pore; Millipore, USA). Prestained protein ladder (Thermo, 26616, USA) was used on the first left lane and the two lanes on the right. Ponceau staining was conducted first to confirm the equal loading and transfer efficacy, then the membranes were incubated with appropriate antibody after blocking with 5%(w/v) skim milk for 1 h. The primary antibody, MAFbx (Anti-Fbx32) (Abcam, ab168372), LC3b (Sigma, L7543), Pan-AKT (Cell Signal Tech, 4691S), Phospho-Akt (Ser473) (Cell Signal Tech, 4060S), FoxO3a (Cell Signal Tech, 2497S), Phospho-mTOR (Ser2448), (Cell Signal Tech, 5536S), mTOR (Cell Signal Tech, 2983S), Phospho-4E-BP1 (Thr37/46) (Cell Signal Tech, 2855S), 4E-BP1 (Cell Signal Tech, 9644S) and glyceraldehyde-3-phosphate dehydrogenase (GAPDH) (Abcam, 181602), were diluted and incubated according to the instructions from the manufacturers, respectively. After washing three times for 10 min each in TBST to remove primary antibody, membranes were incubated with the corresponding second antibody (Cell Signal Tech, USA) for 1 h at room temperature. After washing three times for 10 min each, Super Signal™ West Pico PLUS (Thermo, USA) was used to detect the signal on the membrane. The *ImageJ* software was employed for the quantification of each protein band.

### Quantification and statistical analysis

All quantification and statistical analyses were performed using Prism 9 software. Student’s t-test or one-way ANOVA were used as appropriate. Statistical parameters, numbers of animals and repetitions for each experiment are listed in their associated figure legend. Numbers of repetitions are given in their associated text and/or figure legend.

## Supplementary Information

**Additional file 1: Fig. S1.** Protein expression of the protein homeostasis signaling pathway by western blot in soleus muscles of the hindlimb unloading model mice, fully uncropped and unprocessed images for Fbx32 (MAFbx), GAPDH, LC3B and GAPDH blot are listed. **Fig. S2. **Shows the definition of "3HB-regulated genes" (related to Fig. 3).** Fig. S3.** Shows principal component analysis (PCA) of whole transcriptome data and KEGG pathway of “3HB-regulated genes”, the transcript abundance changes of selected genes (in FPKM) and the results of real-time PCR analysis for *Atg9*, *Vps13d*, and *Chac1* are also listed. **Fig. S4.** Shows protein expression of the Akt/FoxO3a and mTOR/4E-BP1 pathways by western blot in soleus muscles of the hindlimb unloading model mice, fully uncropped and unprocessed images for each blot are listed. **Fig. S5.** Shows relative abundance changes of glycine, neurotransmitters and metabolites of glycolysis and TCA cycle. **Fig. S6.** Shows the blood glucose and blood ketones in the HU model mice.

**Additional file 2: Table S1.** Lists genes in proteostasis gene-set and “3HB-regulated genes”, detailed information for GO and KEGG analysis of “3HB-regulated genes” are also included (related to Fig. 3).

**Additional file 3: Table S2.** Lists raw data of identified metabolites in the targeted and untargeted metabolomic analysis, normalizad data for the metabolites of nucleotide metabolism (related to Fig. 5) and metabolites in Additional file 1: Fig. S5 are also included in sheets.

## Data Availability

The datasets used and/or analyzed during the current study are available from the corresponding author on reasonable request.

## Abbreviations

3HB: 3-Hydroxybutyric acid
4E-BP1: 4E-binding protein 1
Ach: Acetylcholine
ADP: Adenosine diphosphate
AMP: Adenosine monophosphate
ATP: Adenosine triphosphate
FoxO3a: Foxo3a forkhead box O-3
GABA: Gamma-aminobutyric acid
GDP: Guanosine diphosphate
GMP: Guanosine monophosphate
GTP: Guanosine triphosphate
HU: Hindlimb unloading
IA: Indole-acrylic acid
IMP: Inosinic acid
NAAG: N-acetyl-aspartyl glutamate
