# Supplementary material for: Mechanism of reduced muscle atrophy via ketone body (D)-3-hydroxybutyrate
Source: Cell Biosci. 2022 Jun 20;12:94. doi: 10.1186/s13578-022-00826-2 (PMC9208164; doi:10.1186/s13578-022-00826-2)
Supplement: Supplementary file 1 — Additional file 1: Fig. S1. Protein expression of the protein homeostasis signaling pathway by western blot in soleus muscles of the hindlimb unloading model mice, fully uncropped and unprocessed images for Fbx32 (MAFbx), GAPDH, LC3B and GAPDH blot are listed. Fig. S2. Shows the definition of "3HB-regulated genes" (related to Fig. 3). Fig. S3. Shows principal component analysis (PCA) of whole transcriptome data and KEGG pathway of “3HB-regulated genes”, the transcript abundance changes of selected genes (in FPKM) and the results of real-time PCR analysis for Atg9, Vps13d, and Chac1 are also listed. Fig. S4. Shows protein expression of the Akt/FoxO3a and mTOR/4E-BP1 pathways by western blot in soleus muscles of the hindlimb unloading model mice, fully uncropped and unprocessed images for each blot are listed. Fig. S5. Shows relative abundance changes of glycine, neurotransmitters and metabolites of glycolysis and TCA cycle. Fig. S6. Shows the blood glucose and blood ketones in the HU model mice. [file 13578_2022_826_MOESM1_ESM.docx]

Mechanism of Reduced Muscle Atrophy via Ketone Body (D)-3-Hydroxybutyrate

Authors:

Jin Chen^a^, Zihua Li^a^, Yudian Zhang^a^, Xu Zhang^a^, Shujie Zhang^a^, Zonghan Liu^b^, Huimei Yuan^b^, Xiangsheng Pang^b^, Yaxuan Liu^b^, Wuchen Tao^b^, Xiaoping Chen^bc^*, Peng Zhang^c^*, Guo-Qiang Chen^ade^*

Supplementary materials


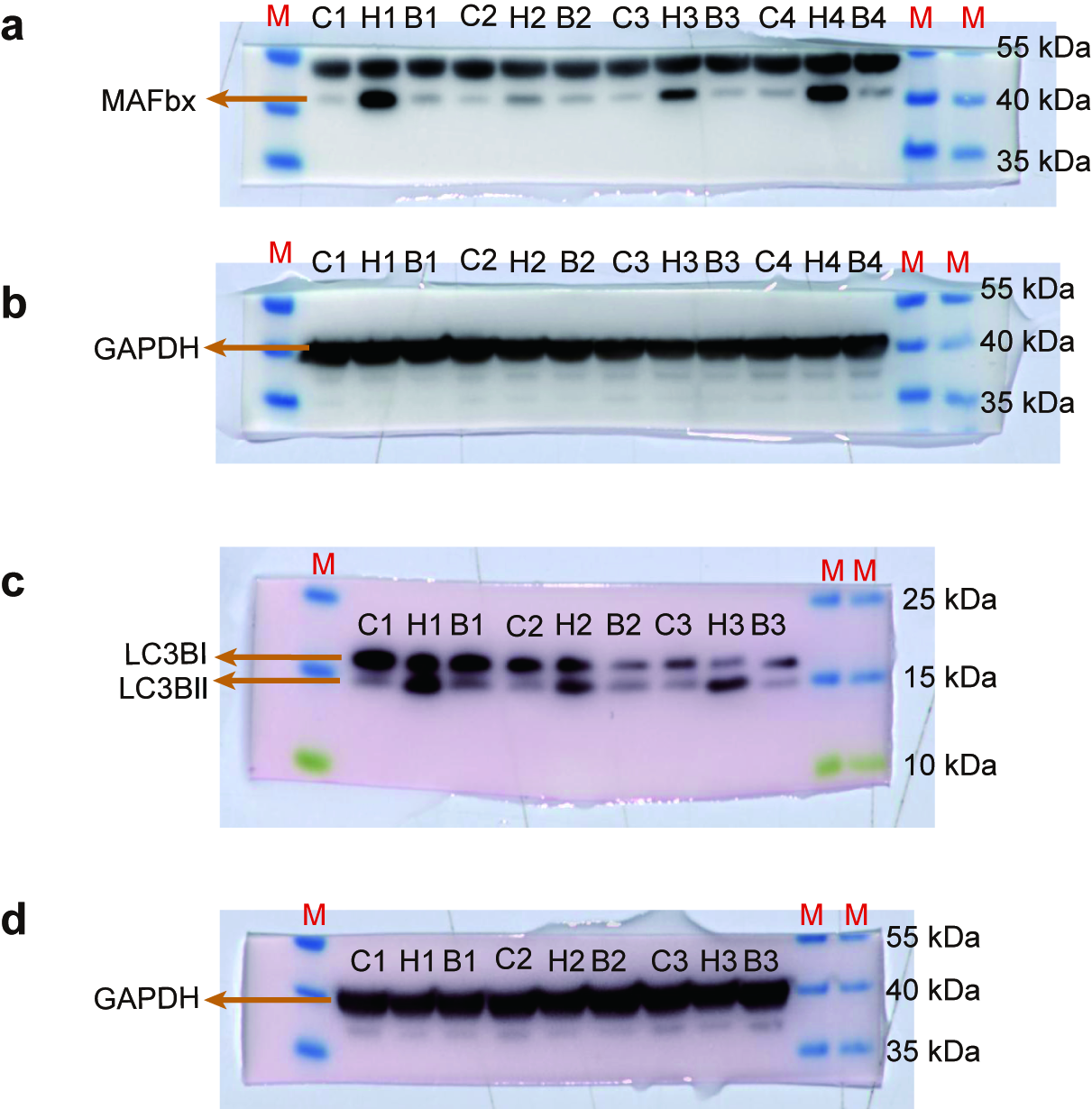


Fig. S1 3HB inhibits the upregulation of ubiquitin-proteasome and autophagy-lysosomal related atrogenes (Related to Fig. 2).

Protein expression of the protein homeostasis signaling pathway by western blot in soleus muscles of mice in C (ground control group, labeled as C1, C2, C3 and C4), H (Hindlimb unloading mouse group, labeled as H1, H2, H3 and H4), and B (hindlimb unloading mice fed with 50 mg/kg/d 3HB group, labeled as B1, B2, B3 and B4). n=3/4 in each group. The first lane on the left lane and the two lanes on the right are protein markers. M: Marker. Orange arrows point to the target band, the sizes of the bands of protein standards (kDa) are labeled on the right side. (a) Ubiquitin protein-Fbx32 (MAFbx), (b) GAPDH, (c) Autophagy protein-LC3B and (d) GAPDH.


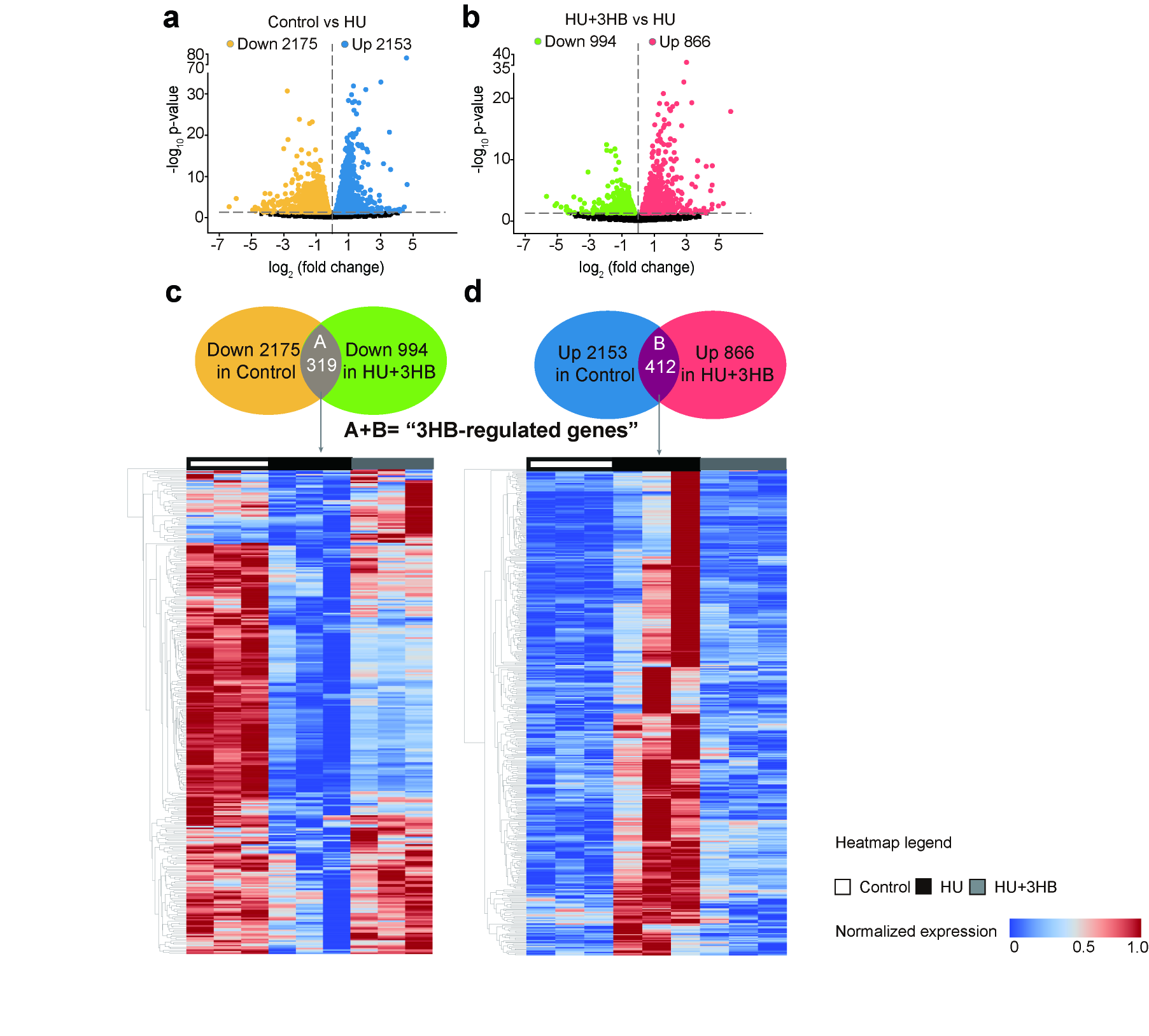


Fig. S2 Definition of “3HB-regulated genes” (Related to Fig. 3).

(a and b) Control vs HU (left) and HU+3HB vs HU (right) volcano plots were constructed using fold change values and p-values. The vertical lines correspond to |log_2_(fold change) | = 0, and the horizontal line represented the p-value = 0.05. The yellow or green/blue or red dots present downregulated/upregulated genes with statistical significance. (c and d) Genes in cluster A (n = 319)/B (n = 412) are both up-regulated (left)/down-regulated (right) in the control group and HB treated group compared with the HU only group. The selected gene sets are displayed with identical colors in (a) and (b), respectively. Cluster A is the overlapped area between 2175 down-regulated genes (Control vs HU) and 994 down-regulated genes (HU+3HB vs HU), while cluster B is the area between 2153 up-regulated genes (Control vs HU) and 866 up-regulated genes (HU+3HB vs HU). Cluster A and B genes are defined as the “3HB-regulated genes”, which behave differently after 3HB treatment under the HU condition compared with the control group. The relative expression abundance (0-1) of these overlapped genes is shown in a heatmap analysis below.


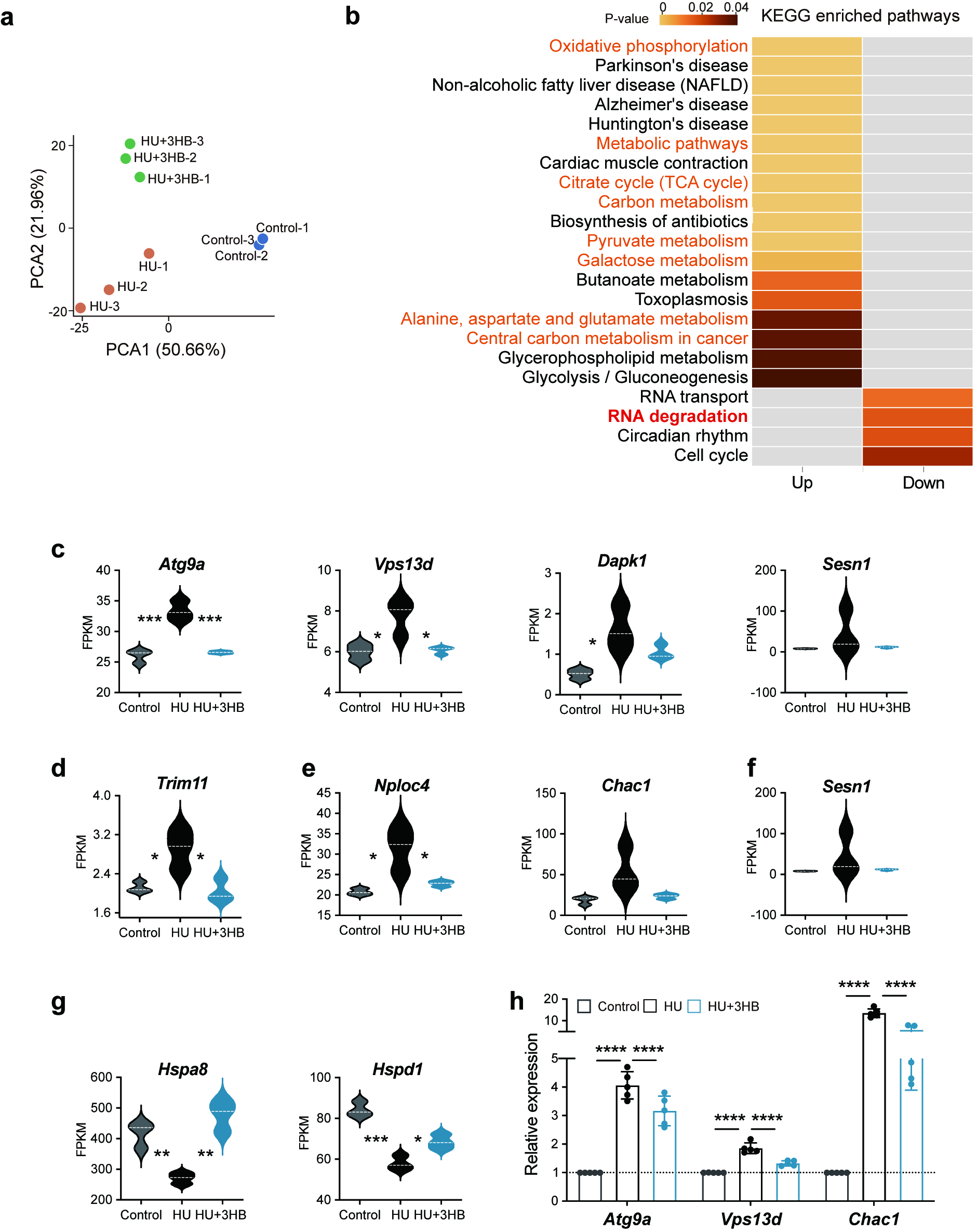


Fig. S3 3HB prevents muscle protein degradation and maintains proteostasis (Related to Fig. 3).

Transcriptomics analysis in soleus muscles of mice in Control (ground control group), HU (Hindlimb unloading treatment group), and HU+3HB (hindlimb unloading mice fed with 50 mg/kg/d 3HB group). n = 3 in each group. (a) All samples were studied using principal component analysis (PCA). The percentage represented on the X-axis and Y-axis accounting for 50.66% (principal component 1) and 21.96% (principal component 2) of the total variation. Control (blue), HU (orange) and HU+3HB (green). (b) KEGG functional annotations of up-and down-regulated DEGs in the 3HB-regulated gene set (defined in Figures 3C and 3D). The gradual color represents the P-value. (c-g) The transcript abundance changes present the selection of differentially expressed genes within: (c) “Autophagy” (*Bnip3*, *Atg9a*, *Vps13d*, *Sesn1*); (d) “Ubiquitin-proteasome system” (*Trim11*); (e) “Unfolded-protein response” (*Nploc4*, *Chac1*); (f) “Antioxidant genes” (*Sesn1*); (g) “Heat shock response” (*Hspa8*, *Hspd1*). FPKM represents gene expression level. (h) Real-time PCR analysis of *Atg9*, *Vps13d*, and *Chac1* in soleus muscles of mice in Control (ground control group), HU (Hindlimb unloading treatment group), and HU+3HB (hindlimb unloading mice fed with 50 mg/kg/d 3HB group).

Error bars are represented as mean ± SD (n = 5). one-way ANOVA was used for comparison between groups.  ****P<0.0001, ***P<0.001, **P<0.01, *P<0.05, compared with HU mouse group.


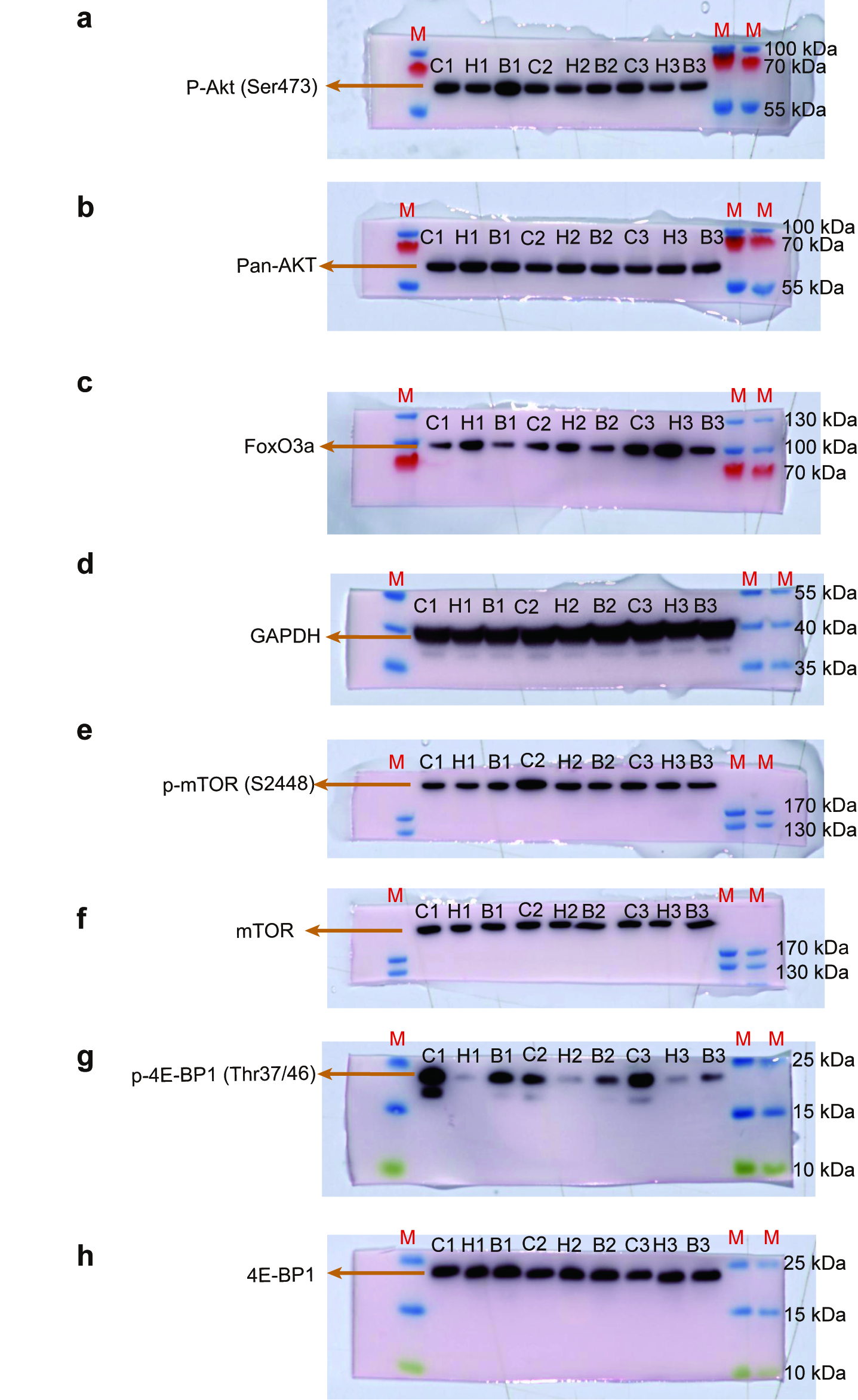


Fig. S4 3HB influences protein metabolism by regulating Akt/FoxO3a and mTOR/4E-BP1 pathways (Related to Fig. 4).

Protein expression of the protein homeostasis signaling pathway studied using Western blot in soleus muscles of mice in C (ground control group, labeled as C1, C2, and C3), H (Hindlimb unloading mouse group, labeled as H1, H2, and H3), and B (hindlimb unloading mice fed with 50 mg/kg/d 3HB group, labeled as B1, B2, and B3). n = 3 in each group. The first lane on the left lane and the two lanes on the right are protein markers. M: Marker. Orange arrows point to the target band, the sizes of the bands of protein standards (kDa) are labeled on the right side.

(a) P-Akt (Ser473), (b) Pan-AKT; (c) FoxO3a, (d) GAPDH, (e) p-mTOR(S2448), (f) mTOR, (g) p-4E-BP1(T37/46) and (h) 4E-BP1.


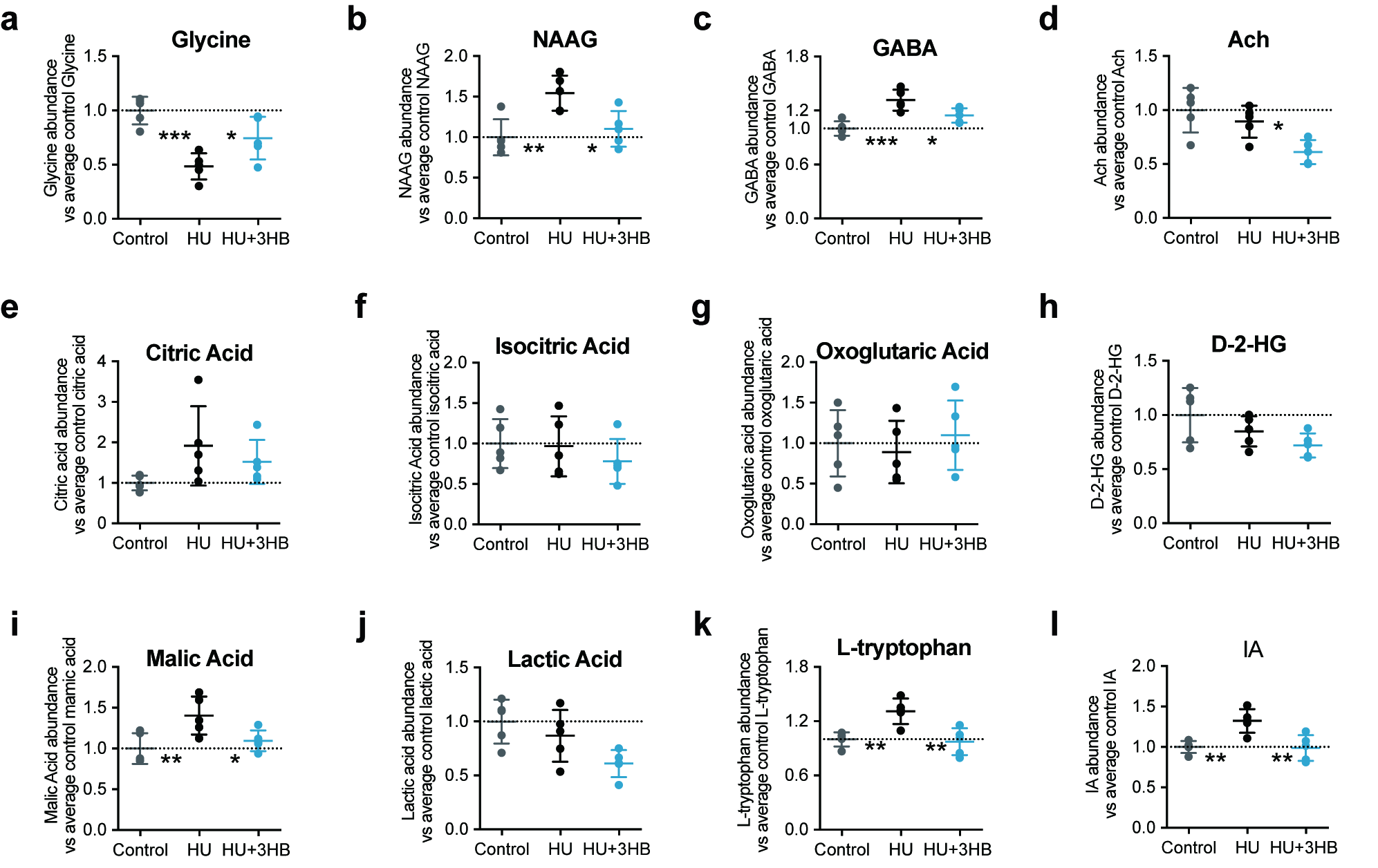


Fig. S5 3HB participates in nucleotide metabolism and reduces uric acid accumulation in atrophied muscles (Related to Fig 5).

Effects of 3HB on skeletal muscle metabolism in soleus muscles of mice in Control (ground control group), HU (hindlimb unloading mouse group), and HU+3HB (hindlimb unloading mice fed with 50 mg/kg/d 3HB group). For each identified metabolite, the raw data of peak area for each sample in the Control, HU and HU+3HB groups were normalized to the average peak area of the control group. (a-l) The relative metabolite abundance for each metabolite among groups: (a) Relative abundance of Glycine. (b) Relative abundance of N-acetylaspartylglutamic acid (NAAG). (c) Relative abundance of gama-aminobutryic acid (GABA). (d) Relative abundance of acetylcholine (Ach). (e) Relative abundance of citric acid. (f) Relative abundance of isocitric acid. (g) Relative abundance of oxoglutaric acid/α-ketoglutaric acid. (h) Relative abundance of D-2-Hydroxyglutarate (D-2-HG). (i) Relative abundance of malic acid. (j) Relative abundance of lactic acid. (k) Relative abundance of L-tryptophan. (l) Relative abundance of indoleacrylic acid (IA). Raw data and normalized data for metabolites above are listed in **Table S2**

Error bars are represented as mean ± SD (n = 5). One-way ANOVA was used for comparison among groups.  ****P<0.0001, ***P<0.001, **P<0.01, *P<0.05, compared with HU mouse group.


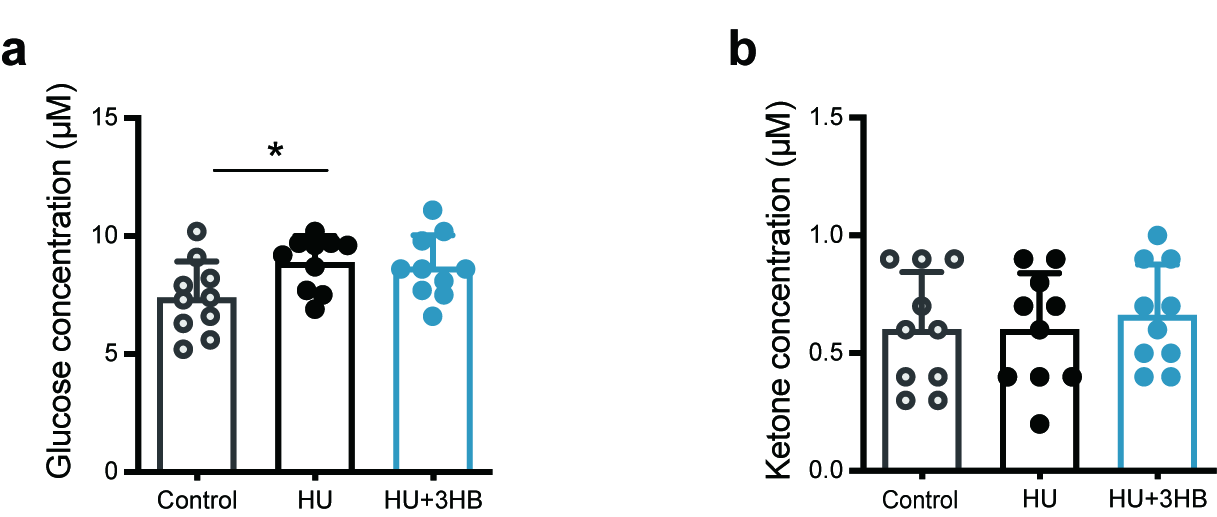


Fig. S6 Blood glucose and blood ketones in the HU model mice.

(a) Blood glucose and (b) blood ketones of mice in Control (ground control group), HU (hindlimb unloading mouse group), and HU+3HB (hindlimb unloading mice fed with 50 mg/kg/d 3HB group) when sacrificed.

Tables 1 and 2 are provided online. Table S1 lists genes in proteostasis gene-set and “3HB-regulated genes”, detailed information for GO and KEGG analysis of “3HB-regulated genes” are also included in the sheets (related to Fig. 3). Table S2 lists raw data of identified metabolites in the targeted and untargeted metabolomic analysis, normalizad data for the metabolites of nucleotide metabolism (related to Fig. 5) and metabolites in Fig. S4 are also included in sheets.
